# GAIN-BRCA: a graph-based AI-net framework for breast cancer subtype classification using multiomics data

**DOI:** 10.1093/bioadv/vbaf116

**Published:** 2025-05-14

**Authors:** Jai Chand Patel, Sushil Kumar Shakyawar, Sahil Sethi, Chittibabu Guda

**Affiliations:** Department of Genetics, Cell Biology and Anatomy, University of Nebraska Medical Center, Omaha, NE 68198, United States; Department of Genetics, Cell Biology and Anatomy, University of Nebraska Medical Center, Omaha, NE 68198, United States; Department of Genetics, Cell Biology and Anatomy, University of Nebraska Medical Center, Omaha, NE 68198, United States; Department of Genetics, Cell Biology and Anatomy, University of Nebraska Medical Center, Omaha, NE 68198, United States; Center for Biomedical Informatics, Research and Innovation, University of Nebraska Medical Center, Omaha, NE 68198, United States

## Abstract

**Motivation:**

Contextual integration of multiomic datasets from the same patient could improve the accuracy of subtype prediction algorithms to help with better prognosis and management of breast cancer. Previous machine learning models have underexplored the graph-based integration, hence unable to leverage the biological associations among different omics modalities. Here, we developed a graph-based method, GAIN-BRCA, using the native features from mRNA, DNA methylation (CpG), and miRNA data as well as the synthesized features from their interactions. GAIN-BRCA computes weightage from miRNA-mRNA and CpG-mRNA interactions to derive a new transformed feature vector that captures the essential biological context.

**Results:**

GAIN-BRCA demonstrates superior performance with an AUROC of 0.98. GAIN-BRCA, with an accuracy of 0.92 also outperformed the existing methods like MOGONET and moBRCA-net with accuracies of 0.72 and 0.86, respectively. Kaplan-Meier survival analysis revealed subtype-specific prognostic genes, including KRAS in Luminal A (*P* value = 0.041), TOX in Luminal B (*P* value = 0.008), and MITF and TOB1 in HER2+ (*P* values = 0.029 and 0.025, respectively). However, no single gene demonstrated a significant survival correlation unique to the Basal subtype. GAIN-BRCA framework, in combination with SHAP, has identified several subtype-specific biomarkers to aid in the development of precision therapeutics for breast cancer subtypes.

**Availability and implementation:**

GAIN-BRCA code is publicly accessible on https://github.com/GudaLab/GAIN-BRCA.

## 1 Introduction

Female breast cancer has now exceeded lung cancer as the most diagnosed cancer, with an estimated 300,000 new cases each year (14.3%), followed by prostate (13.6%), lung and bronchus (11.28%), and colorectal (7.24%) cancers (Siegel *et al.* 2023). Based on the gene expression profiling, breast tumors were subclassified into four groups by Perou *et al.*, which include basal-like, ERBB2-positive (HER2-positive), normal-breast-like, and luminal epithelial/estrogen receptor (ER) positive (Perou *et al.* 2000). This observation was further confirmed by Sorlie *et al.* based on the expression patterns of 534 intrinsic genes in 115 malignant breast cancers (Sorlie *et al.* 2003). Thereafter, Parker *et al.* introduced the PAM50 model, which primarily comprises genes related to hormone receptors, proliferation, myoepithelial, and basal features that are widely applicable in clinical settings (Parker *et al.* 2009). The rationale for this grouping was the variations in the molecular characteristics of tumors, which reflect the discrepancies in gene expression patterns that underlie the breast cancer subtypes (Sorlie *et al.* 2001). While this classification remains a clinical standard, it provides only a partial view of the molecular landscape of breast cancer. These markers do not fully capture the underlying genomic, epigenomic, and transcriptomic heterogeneity that drives tumor behavior, treatment response, and patient outcomes. Multiomics integration, such as combining mRNA expression, DNA methylation, and miRNA profiles, offers a more comprehensive perspective by accounting for complex regulatory layers that influence gene expression and phenotype. Unlike single-modality or marker-based approaches, multiomics data enables the discovery of novel molecular signatures and subtype-specific mechanisms. This broader biological context motivates the need for integrative computational approaches that can extract meaningful patterns across omics layers.

Several studies have used multiomics data from TCGA breast cancer samples that include different types of genomic, epigenomic, transcriptomic, and proteomic data to integrate, train, and test supervised and unsupervised machine learning (ML) models to predict cancer subtypes (Ramazzotti *et al.* 2018, Chen *et al.* 2020, Briscik *et al.* 2024, Shakyawar *et al.* 2024). Most of the multiomics integration strategies are based on either concatenation of molecular features into a feature vector before or after feature selection, or implementation of an autoencoder for feature reduction. Concatenation is the simplest way to process a dataset by horizontally stacking all the omics features together, providing a very vague impression of omics integration without consideration of any biological interactions among them (Ma *et al.* 2016). An R-package *mixOmics* employs the *Diablo* algorithm to make purely linear correlations among the omics datatypes. On the other hand, autoencoder is a neural network-based integration and feature transformation approach, where multiomic features are reduced to a smaller feature representation layer commonly known as the bottleneck. Using this approach, a deep flexible neural forest (DFNForest) was trained on the reduced feature vector extracted from mRNA, miRNA, and methylation data using a stacked autoencoder (Xu *et al.* 2019). In the same context, DeepMO also used an autoencoder for mRNA, methylation, and copy number variation (CNV) data, separately, and transformed features were introduced into the classification layer for breast cancer subtype prediction (Lin *et al.* 2020). MoGCN comprised of transformed mRNA, CNV, and reverse phase protein array features from multimodal autoencoder and patient similarity networks to use with a convolutional graph for patient classification (Li *et al.* 2022).

Recently, in the MOGONET framework, a 3D tensor was formed using a graph convolutional network based on sample similarity networks from mRNA, methylation, and miRNA datasets, individually, which were then passed on to a correlation discovery network for the biomarkers prediction (Wang *et al.* 2021). This approach showed patient correlations across multiomics but failed to address the feature level integration and the underlying biology associated with those features. A recently published methodology called moBRCA-net assigns different weights to omics features based on a self-attention module and individually transforms mRNA, miRNA, and methylation features into interpretable matrices (Choi and Chae 2023). However, this model cannot explicitly understand the dependencies among omics features via a graph network. Along with limited understanding of omics-level correlations and the underlying biology driving these relationships, most of the approaches discussed above also suffer from the autoencoder's hidden black-box functionality. Autoencoders offer a lower-dimensional representation of the data, but it might be challenging to comprehend this representation in terms of its biological significance. Also, autoencoders dealing with high-dimensional data are prone to overfitting, resulting in reduced generalizability for new data (Ma and Zhang 2019, Liu *et al.* 2021). Hence, integration of omics data from multiple platforms such as genomics, transcriptomics, and epigenomics, using a single architecture of autoencoder may not capture the data patterns precisely. Similarly, other models only account for omics associations through unweighted graph networks and do not address the biological relationships among them, which results in a loss of information (Espinoza *et al.* 2020).

In the current study, we implemented a GAIN-BRCA framework for integrating three modalities of breast cancer multiomics data (mRNA, DNA Methylation, and miRNA) by accounting for their feature interdependencies using weighted graph networks. Theoretically, the expression of several mRNAs can be modulated by a single miRNA, while several miRNAs can also modulate a single mRNA (Xu *et al.* 2016, 2018). Previously, miRNA malfunction has reportedly been attributed to the carcinogenesis of several cancers (Lowery *et al.* 2009, Bockmeyer *et al.* 2011, Radojicic *et al.* 2011, Xu *et al.* 2020). Similarly, the methylated status of CpG islands also modulates gene expression in the neighboring loci (Morgan and Marioni 2018, Song *et al.* 2018, Yi *et al.* 2021, Silva *et al.* 2022). Hence, both methylation and miRNA levels could regulate the expression profile of the associated mRNAs, leading to cancer development and progression. Due to these dependencies, the proportionate flow of biological information and its correlations at the molecular level must be preserved while formulating objective functions in machine learning models. To meet these requirements, we quantified the biological associations among all miRNA-mRNA and CpG-mRNA pairs using integrated weighted graphs and used them to train the ML models for subtype prediction. We conducted feature importance analysis to determine the key features influencing model predictions, followed by downstream investigations to perform survival analysis and identify unique marker genes associated with breast cancer subtypes.

## 2 Methods

### 2.1 Datasets

**Figure 1.**
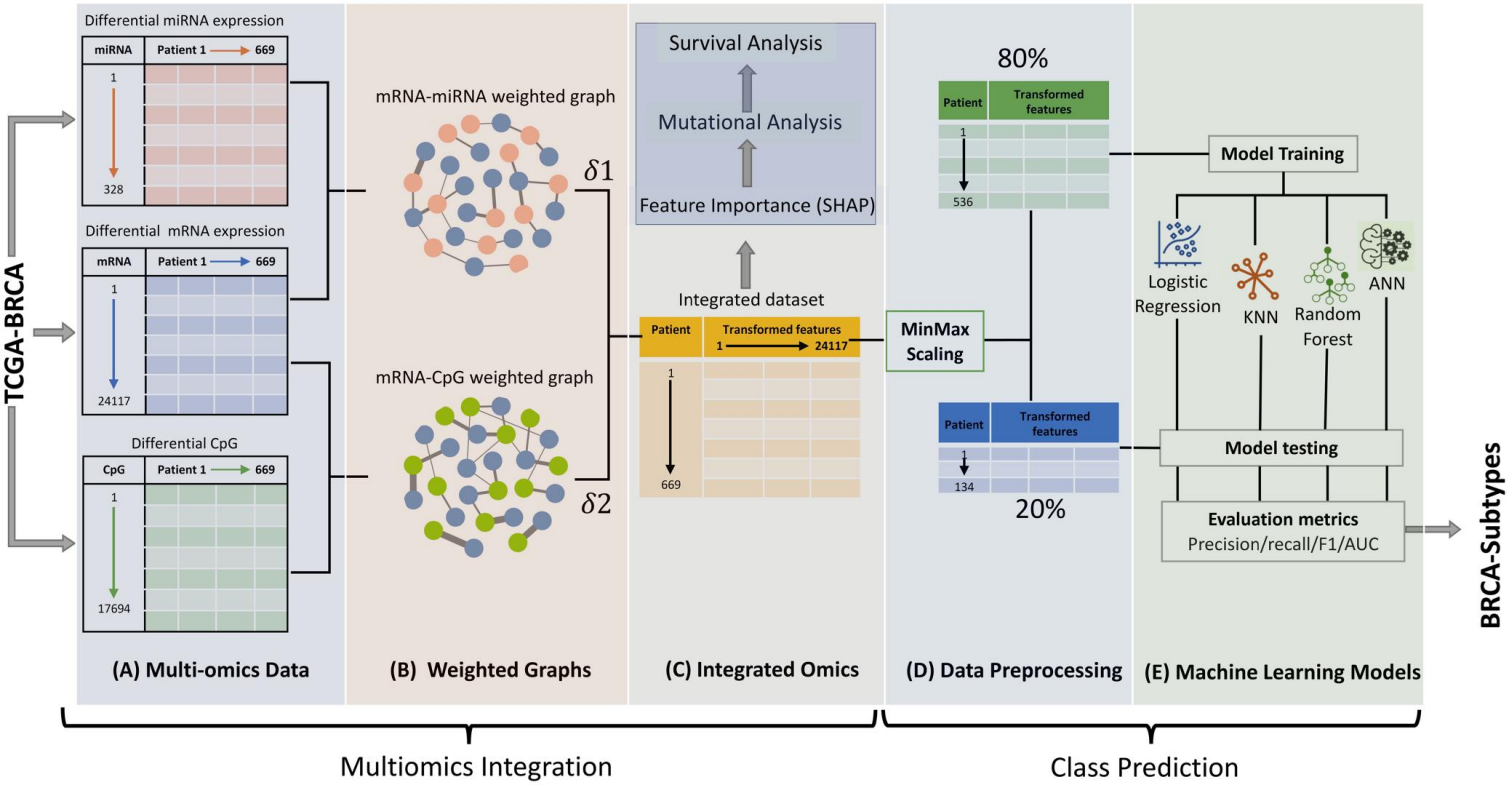
Depiction of GAIN-BRCA flow. (A) TCGA-BRCA differentially expressed (DE) miRNA (orange), DE mRNA (blue), and differential CpG methylation (green) were processed from the same patient cohort. (B) mRNA-miRNA and mRNA-CpG weighted graphs were constructed using interaction values between corresponding matched omics data. (C) These graphs were used to derive the modulating factors δ1 and δ2 from the equation proposed in the methods, and all transformed features were obtained (dark yellow matrix). Important features were extracted from the integrated dataset using Shapley Additive exPlanations (SHAP). (D) Data were scaled and split into training and test datasets (E) Machine learning models were trained on integrated datasets to predict subtypes.

Breast cancer datasets were retrieved from TCGA (https://portal.gdc.cancer.gov/) using the R/Bioconductor library *TCGAbiolinks*, which allows users to design queries for desired datasets and send them to the Genomic Data Commons data portal through the application programming interface (Gentleman *et al.* 2004, Colaprico *et al.* 2016). RNA-Seq and miRNA-Seq data were queried under transcriptomic profiling, while methylation data were queried under the DNA methylation category of TCGA-BRCA to fetch the data in RStudio (Posit Team 2023). The three directories, i.e. mRNA, miRNA, and DNA methylation (CpG) containing, 1059 patients’ sample files in each were stored, and the files for each omic were merged to get a single file for each omic, separately. Deidentified clinical information for breast cancer was obtained separately from TCGA. Figure 1 depicts the workflow for the overall methodology used in the current study.

### 2.2 Data preprocessing

In this study, we filtered out miRNAs, mRNAs, and CpG probes containing more than 20% zeros or “NA” symbols across all the patients in their corresponding matrices. We also excluded patient samples with more than 20% zeros or NAs in each single-omic data matrix. Finally, we retained only those patient samples that contained all three omic datasets after initial filtering, making the final count of 669 patients (Table 1). miRNA and mRNA data were further normalized using the *DESeq2* package, and final matrices were generated. To further process methylation data, we used the R package, VIM, for imputing missing *β* values in the matrices, followed by *β* mixed-integer quantile normalization using the *ChAMP* pipeline (Morris *et al.* 2014, Kowarik and Templ 2016). We included only CpGs which are 0–1500 bases upstream of the transcription start sites, as described in Mishra et al. (Mishra and Guda 2017, Mishra *et al.* 2020). The summary of the processed data, including several samples and features that were used as input for the model training, is given in Table 1. The molecular subtype information for 669 BRCA samples was collected from TCGA metadata files. The distribution of BRCA subtypes in our dataset was imbalanced, therefore, we also augmented the minority class samples using the Adaptive Synthetic Sampling Approach for Imbalanced Learning (ADASYN) and achieved a balanced representation across classes (He and Garcia 2009). ADASYN adaptively creates more samples in regions where the data were sparse, focusing on minority class examples near decision boundaries based on density distribution to help improve classifier performance by balancing class distributions (Table 2).

**Table 1.** Details of single-omic features after pre-processing.^a^

Single-omic type	No. features	Workflow type
mRNA	24 117	STAR—Counts
miRNA	328	BCGSC miRNA PROFILING
DNA Methylation	17 694	SeSAMe Methylation *Β* Estimation

Number of patient samples containing all three omic data are 669.

**Table 2.** The number of samples in the PAM50-based intrinsic BRCA subtypes from the original and augmented datasets.

BRCA Subtype	Original samples	Training set after split	Training set after augmentation	Test set
Luminal A	393	354	366	39
Luminal B	124	111	396	13
HER2+	38	34	400	4
Basal	114	103	374	11
Total	669	602	1536	67

### 2.3 Omics data integration strategies

Cancer is often an outcome of altered signaling mechanisms coordinated by proteins translated from expressed mRNA that are regulated by miRNA and DNA methylation. Hence, a method that incorporates the weighted interrelationships between miRNA-mRNA and CpG-mRNA into its objective function would make the algorithm more biologically intuitive. In our multiomics data integration strategy, we introduced two mRNA-modulating factors: *δ1* and *δ2* for the effects of associated miRNA(s) and CpGs, respectively. These two factors describe the quantitative implications of miRNA and CpG on mRNA expression. Theoretically, interacting miRNAs are responsible for lowering mRNA expression, as indicated by Equation (1), where the sum of the expression of all miRNAs was set exponentially inversely proportional to the expression of the associated mRNA.


(1) $$\mathrm{mRNA}\propto \frac{1}{e^{\alpha *\log2(\sum \mathrm{miRNA}\_\mathrm{expression})}}$$

∑miRNA_expression is the sum of the expressions of all miRNAs that interact with a specific mRNA. *α* is a hyperparameter which can be set empirically. As Equation (1) depicts the exponential decay effects of miRNAs on the expression of associated mRNAs, the *α* regularizes the narrowness of the curve. Technically, a higher *α* will generate a narrower curve with less observable transformed expression values between two mRNAs. Thus, we empirically set a lower value of *α* (equal to 1.5). Further, we modified Equation (1) by incorporating the miRNA-mRNA interaction score (IS), which was generated by normalizing weighted context scores from TargetScan (Agarwal *et al.* 2015). In this analysis, we included 34,013 miRNA-mRNA interacting pairs, which were common in (pairs with high and moderate confidence, weighted context score <−0.2) and mintRULS (all predicted pairs with strong and moderate interactions) (Shakyawar *et al.* 2022). We considered common pairs from these tools to build higher confidence in choosing computationally predicted interactions between miRNAs and mRNAs. The stronger the IS, the greater its effect on mRNA downregulation will be. This analogy can be written as Equation (2).


Equation (2) $$\mathrm{mRNA}\propto \frac{1}{\mathrm{IS}(\mathrm{miRNA}-\mathrm{mRNA}}$$

Combining Equations (1), we get Equation (3), which defines the modulating factor *δ*1, representing average modulation by all miRNAs on *n* number of mRNAs.


(3) $$\delta 1=\frac{1}{n}(\sum_{i=1}^{n}\frac{1}{{\mathrm{IS}({\mathrm{miRNA}}_{\;}-{\mathrm{mRNA}}_{i})\;\times \;e}^{\alpha *\log2(\mathrm{expr}[\mathrm{miRNA}])}})$$

Similarly, to implement modulating factor *δ2*, we assume that cumulative methylation levels, represented by the sum of *β* values of CpG sites located within 0 to 1500 base pairs (bp) upstream of an mRNA transcription start site are inversely proportional to the level of corresponding mRNA expression, as indicated by Equation (4).


(4) $$\mathrm{mRNA}\propto \frac{1}{e^{\beta *\log2(\sum \mathrm{CpG})}}$$

∑ CpG refers to the sum of the *β* values of all CpGs associated with a specific mRNA. Unlike miRNA-mRNA interactions, we have no CpG-mRNA interaction score. So, we can directly derive *δ2* from Equation (5).


(5) $$\delta 2\;=\frac{1}{n}(\sum_{i=1}^{n}\frac{1}{e^{\beta *\log2(\mathrm{CpG})}})$$

β in (5) has a similar interpretation as *α* in Equation (3) and was set to 1.5 in this study. We believe that *δ1* (miRNA-induced modulating factor) and *δ2* (CpG-induced modulating factor) affect the expression of associated mRNA. To observe the combined effect of both miRNA and CpG, we took an average of both *δ1* and *δ2* to calculate the final modulating factor *δ* [Equation (6)].


(6) $$\delta \;=\frac{\delta 1+\delta 2}{2}$$

We interpret *δ* as the final magnitude of impact from miRNA and CpG on mRNA expression. This *δ* factor multiplied by the respective mRNA expression value gives a transformed value of mRNA, as illustrated in Equation (7).


(7) trans(mRNA) = δ × expr(mRNA)

where expr(mRNA) and trans(mRNA) represent original and transformed expression values of mRNA, respectively.

### 2.4 GAIN-BRCA model design

The GAIN-BRCA multiomics integration model calculates and quantitatively incorporates the impact of miRNA and CpG on mRNA expression. This model requires the mRNA and miRNA expression, methylation *β* values, and mRNA-miRNA interaction scores to determine the transformed *δ1* and *δ2* values. Equation (3) takes expression values of only one mRNA at a time, along with its associated miRNAs and interaction scores between them, and repeats this process across all mRNAs to calculate the modulating factor, *δ*1. Similarly, the mRNA associated with CpG *β* expression was used in Equation (5) to determine the modulating factor, *δ*2. We performed experiments by taking equal weight combinations for *δ*1 and *δ*2 to generate the final modulating factor *δ*, as shown in Equation (6). The modulating factor *δ* was multiplied by the respective mRNA expression score to obtain the final transformed matrix as shown in Equation (7). The features from the final transformed matrix were used for building machine learning models. We compared the GAIN-BRCA model with different multiomics feature integration methods, such as MOGONET and moBRCA-net and employed machine-learning models for subtype prediction. These two methods are based on graph-based data integration similar to our method.

### 2.5 Concatenation-based ML model predictions

We first concatenated the mRNA, miRNA, and DNA methylation datasets to create a single integrated multiomics dataset. Each dataset has a p×n dimensions in which p and n denote the samples and features, respectively. The dataset was split into training and test sets in an 80:20 ratio. The models were trained using stratified five-fold cross-validation on the logistic regression (LR), K-nearest neighbor (KNN), random forest (RF) from scikit-learn, and artificial neural network (ANN) from *Keras* modules (Ho 1995, Mucherino *et al.* 2009, Pedregosa *et al.* 2011, Chollet *et al.* 2015,  Chen and Guestrin 2016). The LR model was used as a multiclass prediction problem with one versus rest argument, keeping other arguments as default. We did not modify the implementation of the RF, whereas, for KNN, the number of neighbors was set to 4. The sequential architecture for the ANN gives the best results with three hidden layers and one last layer, out of experiments performed with 2 to 10 hidden layers. We used the activation function ReLU and softmax for the hidden and last layers, respectively. We tested Adagrad, Adamax, and Adam as optimizers, where Adam came out on the top. As our objective is a multiclass problem, we applied sparse categorical cross-entropy as a loss function. We used precision, recall, accuracy, and F1-score as performance metrics for model evaluation. A comparison of the performance of different models was done using the area under the receiver operating characteristic curve (AUROC) metric.

### 2.6 Autoencoder-based ML model predictions

Autoencoder, a well-known multiomics integration approach based on the feature transformation approach, was used to compare with GAIN-BRCA (Liou *et al.* 2014). Autoencoder has two parts: first, the encoder compresses the original data (x) and stores it as a reduced feature vector (f), while the second, the decoder tries to regenerate the original dataset ($\tilde{x}$) from the reduced feature vector, minimizing the loss of data (l). The reduced feature vector (f) representing the original dataset preserves the discriminative features to classify the datasets. The autoencoder took the multiomics dataset as a concatenated matrix and compressed the dimensions of the original data into a reduced feature vector, which was used in machine learning models. Again, LR, KNN, RF, and ANN were trained using a reduced feature vector from the autoencoder.

### 2.7 GAIN-BRCA–based predictions

GAIN-BRCA–based multiomics data integrated feature matrix was used to train the ML models that include LR, KNN, RF, and ANN. The performance of ML models trained on concatenated, autoencoder, and GAIN-BRCA based multiomics data integration approaches was compared. For comparison, we also generated a set of ML models to evaluate the performance when trained on data from only a single-omic modality relative to multiomic integration approaches described above.

### 2.8 Identification of explainable features

Unlike autoencoders, which compress input data into latent representations that are often difficult to interpret, GAIN-BRCA enables the interpretability by preserving the biologically meaningful features, which allows us to identify critical subtype specific genes that drive model predictions. This transparency facilitates feature importance analysis and enhances our understanding of how individual features contribute to classification decisions. In cancer genomics, interpretability is vital for the reliance on model’s output, validation, and hypothesis generation for downstream research. By enabling insights into subtype-specific gene contributions, GAIN-BRCA supports hypothesis generation and enhances clinical relevance, offering both predictive power and transparency beyond conventional black-box models. Shapley additive explanations (SHAP) is one of the best tools to extract the essential features contributing individually to the target prediction (Lundberg and Lee 2017). A Python package shap was used to perform the underlying contributions and importance of omic features in model predictions. The training data were fit into the random forest classifier model and called by the shap. Tree explainer to get a list of the best discriminative features associated with different breast cancer classes. These SHAP features may be involved in the cause and advancement of cancer, therefore, we used Ingenuity Pathway Analysis (IPA) to extract the upstream regulators and downstream molecules for the SHAP features and merged them all together to get a single gene list. These genes were searched in the TCGA-BRCA cohort to check out the number of associated patients with mutational profiles. If a gene has at least one deleterious or probably damaging mutation in the TCGA-BRCA patient cohort, such genes are functionally more significant in breast cancer biology. We identified genes with at least one such mutation using the Sorting Intolerant From Tolerant (SIFT) (Ng and Henikoff 2003) score between 0.0 and 0.05 (labeled as deleterious) and PolyPhen (Flanagan *et al.* 2010) score between 0.85 and 1.0 (labeled as probably damaging). Thereafter, the haplo-insufficiency scores for all the genes were extracted from DECIPHER (Bragin *et al.* 2014). It is a genomic database that facilitates the interpretation of rare genetic variants, particularly copy number variations and single nucleotide variants, associated with developmental disorders. It provides tools for visualizing patient data, identifying pathogenic variants, and correlating genetic changes with clinical phenotypes. A haploinsufficiency score indicates a gene's reduced functionality or aberrant dosage of its product caused by the loss of one copy, reflecting its sensitivity to dosage changes. We mapped all the explainable features (genes) in different subtypes of the TCGA-BRCA cohort and obtained unique features for each subtype.

### 2.9 Subtype-specific prognostic gene evaluation

To evaluate the prognostic relevance of subtype-specific genes identified for each breast cancer subtype, a Kaplan-Meier (KM) survival analysis was conducted. For each subtype, patient survival data were analyzed in relation to the expression levels of the identified genes. Statistical significance of survival differences was assessed using the log-rank test, with a threshold of *P* < 0.05 considered significant. Subtype-specific survival curves were generated, and associations between gene expression and survival outcomes were visualized. The analysis was performed for all subtypes: Luminal A, Luminal B, HER2+, and Basal, using dedicated survival analysis tools and R packages. This approach aimed to identify genes with potential prognostic value and their implications for clinical outcomes in a subtype-specific manner.

## 3 Results and discussion

### 3.1 Performance of single-omic approach

**Figure 2.**
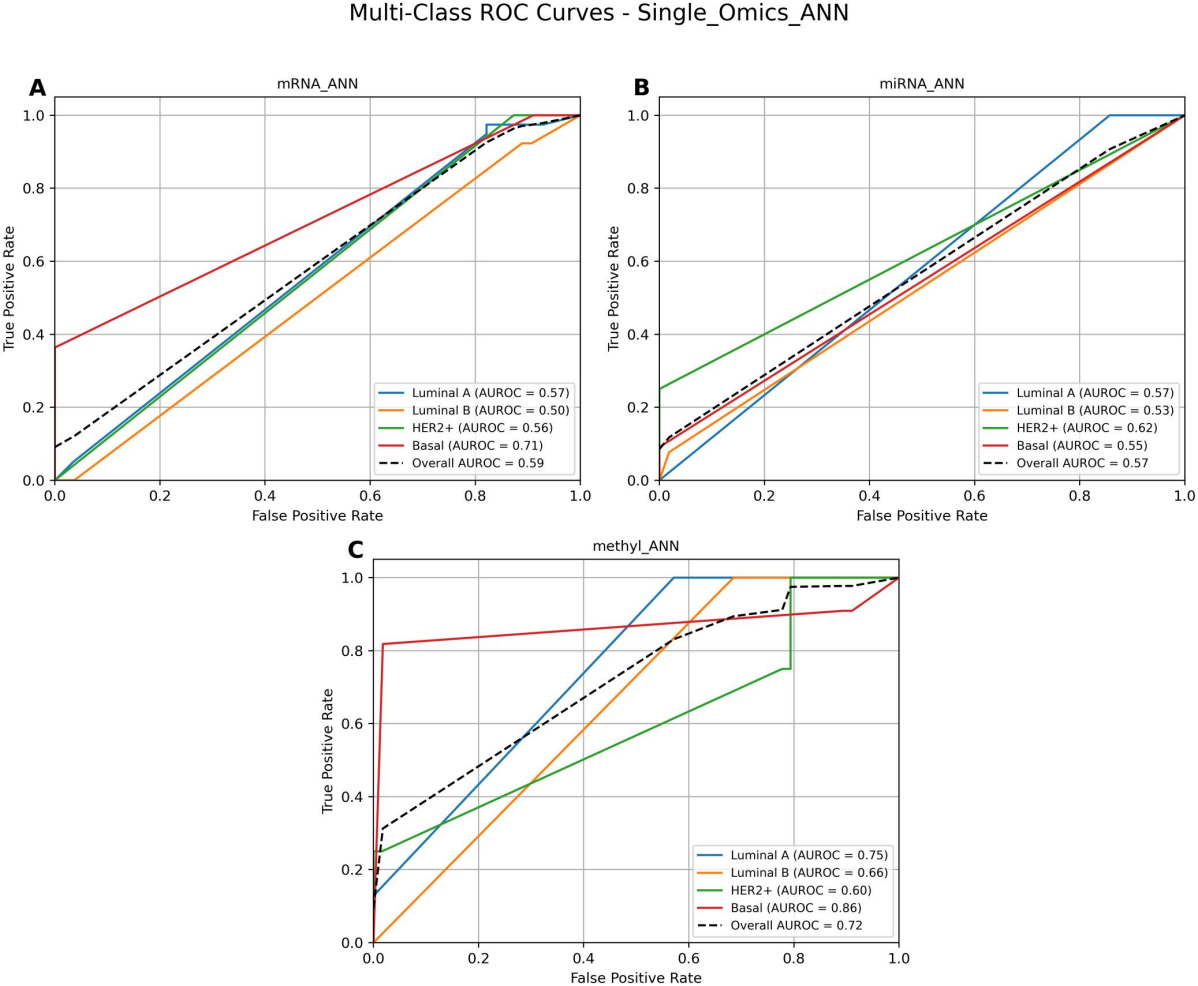
ROC curves illustrate the performance of ANN models using single-omic approaches for predicting breast cancer subtypes: Luminal A, Luminal B, HER2+, and Basal- are presented for each omic dataset: (A) mRNA, (B) miRNA, (C) DNA methylation. Overall AUC for the model as well as subtype-specific AUCs were presented.

We evaluated the effectiveness of individual omics data for breast cancer subtype classification by training a separate model on each omic dataset, i.e. mRNA, miRNA, and DNA methylation, using ANN. The ANN model was trained and cross-validated for each dataset to gauge its discriminative power for subtype prediction (Fig. 2). Among these, the DNA methylation dataset exhibited the highest predictive capability, with an overall AUROC of 0.72 (Fig. 2C), suggesting that DNA methylation features may offer valuable insights for distinguishing breast cancer subtypes. The mRNA dataset followed with a moderate overall AUROC of 0.59 (Fig. 2A), while the miRNA dataset showed the lowest performance, with an overall AUROC of 0.57 (Fig. 2B). It is noteworthy that the AUROC values of all individual subtypes in the DNA methylation model are the highest among their counterparts in other omics data models, showing that the former data modality has the best discriminative power. These results demonstrate different predictive strengths of single-omic data models and underscore the need to integrate different modalities of omics to build robust and comprehensive models to enhance the classification accuracy by capturing a broader range of biological information and their interrelationships.

### 3.2 GAIN-BRCA multiomics integration framework

GAIN-BRCA is a multiomics integration approach for the classification of breast cancer subtypes. It starts with preprocessing steps that include determination of the effects of miRNA and CpG on mRNA expression using weighted graph networks, which will be combined for each patient to build a transformed matrix. The alteration effect in mRNA expression due to miRNA and CpG was denoted as *δ*1 and *δ*2, respectively. To determine the contribution of miRNA-mRNA and CpG-mRNA interactions toward subtype prediction, we tested various weight ratios of *δ*1 and *δ*2 modulating factors, respectively, as 1:3, 2:3, 3:1, 3:2, and 1:1 to calculate the final modulating factor *δ*. Thus, the modulation factor *δ* was multiplied by the respective mRNA expression values to get the final transformed matrix. Different neural network models were trained and tested on all weight combinations of *δ*1 and *δ*2 as mentioned above. We observed that the equal weight combination (1:1) of *δ*1 and *δ*2 yielded the best accuracy using the deep feed-forward neural network model. In all the future experiments, we used equal weight combinations of *δ*1 and *δ*2.

### 3.3 Evaluation of all integration approaches using current datasets

Because GAIN-BRCA’s multiomics data integration strategy is based on weighted graph networks and biologically intuitive compared with the existing strategies, such as concatenation-based and autoencoder-based approaches, we also tested the performance of the latter strategies by consistently using the same datasets and ML tools used for developing GAIN-BRCA. Hence, these results will provide a direct reference to evaluate the performance of GAIN-BRCA. To maintain consistency across all models, we performed a stratified 10-fold cross-validation and used the overall AUROC as the primary metric for comparison (Table 3).

**Table 3.** Summary of AUROC results including precision, recall and F1 for various single-omic and multiomic integration approaches in combination with various Machine Learning Models across different breast cancer subtypes: Luminal A, Luminal B, HER2+, and Basal.^a^

Integration approaches	ML models	Metrics	Luminal A	Luminal B	HER2+	Basal	Overall AUROC	Accuracy
Concatenation	LR	Precision	0.7292	0.5714	0	0.75	0.86	71.64
		Recall	0.8974	0.3077	0	0.8182		
		F1	0.8046	0.4	0	0.7826		
	KNN	Precision	0.68	0.4286	0	0.6667	0.72	64.18
		Recall	0.8718	0.2308	0	0.5455		
		F1	0.764	0.3	0	0.6		
	RF	Precision	0.6731	0	0	0.5	0.71	61.19
		Recall	0.8974	0	0	0.5455		
		F1	0.7692	0	0	0.5217		
	ANN	Precision	0.717	0.5	0	1	0.94	74.63
		Recall	0.9744	0.1538	0	0.9091		
		F1	0.8261	0.2353	0	0.9524		
Autoencoder	LR	Precision	0.6842	1	0	1	0.85	73.13
		Recall	1	0.0769	0	0.8182		
		F1	0.8125	0.1429	0	0.9		
	KNN	Precision	0.5909	0	0	1	0.52	59.7
		Recall	1	0	0	0.0909		
		F1	0.7429	0	0	0.1667		
	RF	Precision	0.7	0.2857	0	0.7	0.73	65.67
		Recall	0.8974	0.1538	0	0.6364		
		F1	0.7865	0.2	0	0.6667		
	ANN	Precision	0.8	0.6667	0.8	0.9091	0.92	80.6
		Recall	0.9231	0.3077	1	0.9091		
		F1	0.8571	0.4211	0.8889	0.9091		
GAIN-BRCA	LR	Precision	0.9048	0.8	1	0.8123	0.95	88
		Recall	0.9744	0.6667	0.75	0.6856		
		F1	0.9383	0.7273	0.8571	0.7659		
	KNN	Precision	0.7222	1	1	1	0.85	77
		Recall	1	0.0833	0.25	0.9167		
		F1	0.8387	0.1538	0.4	0.9565		
	RF	Precision	0.7843	0.75	0	0.9167	0.9	80.6
		Recall	1	0.25	0	1		
		F1	0.8791	0.375	0	0.9565		
	**ANN**	**Precision**	**0.9286**	**0.8333**	**1**	**1**	**0.98**	**92**
		**Recall**	**0.975**	**0.7692**	**0.6667**	**1**		
		**F1**	**0.9512**	**0.8**	**0.8**	**1**		
GAIN-BRCA Augmented	LR	Precision	0.8372	0.7778	1	0.8333	0.93	83.5
		Recall	0.9231	0.5385	0.75	0.9091		
		F1	0.878	0.6364	0.8571	0.8696		
	KNN	Precision	0.7812	0.375	0.3333	0.6154	0.74	61.1
		Recall	0.641	0.4615	0.5	0.7273		
		F1	0.7042	0.4138	0.4	0.6667		
	RF	Precision	0.9032	0.4762	0.8	1	0.86	77.6
		Recall	0.7179	0.7692	1	0.9091		
		F1	0.8	0.5882	0.8889	0.9524		
	ANN	Precision	0.8333	0.7778	0.6667	0.9	0.93	82
		Recall	0.8974	0.5385	1	0.8182		
		F1	0.8642	0.6364	0.8	0.8571		

a All models were built using multiomics datasets that include mRNA, miRNA, and DNA methylation data.

The results obtained from GAIN-BRCA with ANN are shown in bold.

### 3.4 Performance of concatenation-based models

One of the simplest multiomics data integration approaches used with ML methods is concatenation, where features from multiple data modalities, such as mRNA, miRNA, and DNA methylation, are combined into a single matrix. In our study, this resulted in a matrix with a dimension of 669 (patients) × 42,139 (all features). We applied various machine learning models, including LR, KNN, RF, and ANN, to this concatenated dataset. The ANN model performed best with an AUROC of 0.94 and an accuracy of 74.63%, with high recall and F1 scores for Luminal A and Basal subtypes, indicating its effectiveness in leveraging combined omics features (Supplementary Fig. S1). Nevertheless, LR came very close to ANN with an overall AUROC of 0.86 and accuracy of 71.64%, while RF and KNN with AUROCs of 0.71 and 0.72, respectively, underperformed in this comparison (Table 3). The concatenation approach, while straightforward, does not account for the complex interactions between different molecular data modalities, potentially limiting its ability to fully capture the biological context of multiomic datasets.

### 3.5 Performance of autoencoder-based models

We also tested the autoencoder approach, which compresses high-dimensional data into lower-dimensional transformed feature vectors. These vectors were then used to build LR, KNN, RF, and ANN models. In this case, ANN performed the best with an overall AUROC of 0.92 and an accuracy of 80.6% (Supplementary Fig. S2) compared to LR, RF, and KNN models (Table 3). It showed balanced performance across all the subtypes, including strong F1 scores for HER2+ (0.88) and Basal (0.90), indicating that the nonlinear relationships between different data modalities are better captured with autoencoders. In addition, the autoencoder-LR (AUROC of 0.85 and accuracy of 73.13%) also performed competitively, slightly lower than concatenation-LR (AUROC of 0.86 and accuracy of 71.64%) model, but with better precision for Luminal B and Basal. However, autoencoder-KNN showed a poor performance compared to concatenation-KNN with an AUROC of 0.52 (Table 3). The poor performance of the KNN model in this instance may be attributed to the prior compression of the dataset using an autoencoder. When the data are compressed, some important details could be lost, making it harder for the KNN to pick up on key patterns needed for accurate results. The RF model only showed modest improvement in its performance on concatenated data. Although autoencoders improved feature representation by capturing non-linear relationships, they still could not model the intricate interactions between different omics layers explicitly, resulting in lower accuracy compared to the concatenation-based models.

### 3.6 Performance of GAIN-BRCA-based approach

**Figure 3.**
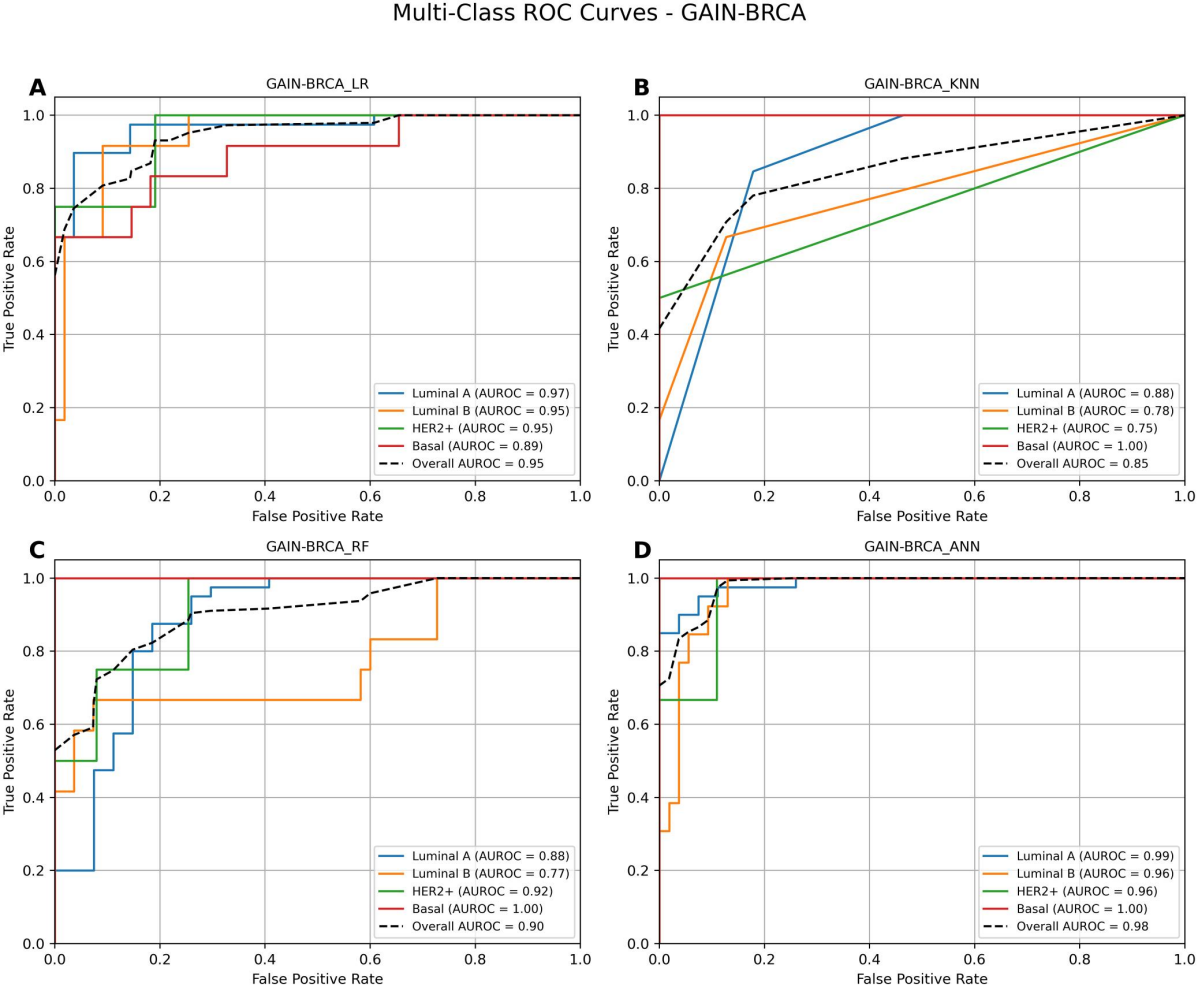
Performance of GAIN-BRCA based integration models. This panel displays the ROC curves for the GAIN-BRCA integration method with (A) logistic regression (LR), (B) K-nearest neighbor (KNN), (C) random forest (RF), and (D) artificial neural network (ANN). ANN performs exceptionally well for all subtypes, achieving an AUROC of 0.98 or higher for Luminal A, Luminal B, HER2+, and Basal. Similarly, LR shows strong results, particularly for the Luminal A subtype, with an AUROC of 0.97. RF also performs well for Basal but struggles with the Luminal B subtype. KNN, in contrast, shows lower AUROC values across all subtypes except Basal. Overall AUC for the model as well as subtype-specific AUCs were presented.

GAIN-BRCA implements a novel graph-based integration framework that is designed to preserve and utilize the intricate relationships between different multiomic data modalities. To ensure a thorough evaluation, we selected LR, RF, KNN, and ANN to build models using the integrated transformed matrix. ANN and LR were chosen because they demonstrated the best predictive performance in the concatenation and autoencoder approach, respectively, but we also assessed the other models for a more comprehensive comparison. Results showed that GAIN-BRCA-based ML models consistently outperformed respective models from concatenation-based and autoencoder-based methods, regardless of the ML model used, indicating the superiority of graph-based fusion in capturing nuanced patterns (Table 3). Among all models, ANN achieved the highest AUROC score of 0.98 and an accuracy of 92% with GAIN-BRCA yielding near perfect F1 scores for Luminal A (0.95), Basal (1.0), and notably strong results for the HER2+ and Luminal B subtypes, making it the most effective model for this framework (Fig. 3D). Similarly, RF achieved an AUROC of 0.95 and accuracy of 88% with GAIN-BRCA (Fig. 3C), which was significantly higher than the RF models using concatenation (AUROC: 0.71) or autoencoder (AUROC: 0.73) methods, showing its capacity to handle the complex relationships captured by the graph-based approach. Similar improvement was observed in the LR models with GAIN-BRCA (Fig. 3A). On the other hand, KNN, which typically underperforms in these types of tasks, showed moderate improvement with GAIN-BRCA, reaching an AUROC of 0.85 and accuracy of 77%, outperforming its earlier performances, substantially (Fig. 3B). The significant gains observed with LR, RF, and KNN further underscore the value of GAIN-BRCA as an advanced integration strategy. Our approach demonstrates a significant advancement in multiomics data integration, as traditional methods like concatenation and autoencoders often overlook the complex interactions between omics layers. GAIN-BRCA not only boosts predictive accuracy, but also improves the interpretability of results by maintaining the biological relationships among omics features, offering a more insightful approach to breast cancer subtype prediction across a range of models.

### 3.7 Comparison with existing models

To test the performance of GAIN-BRCA with ANN and augmented data, we compared our method with two other popular methods, MOGONET and moBRCA-net. While both methods use graph-based multiomics integration strategies, MOGONET employs graph-based interactions among multiomics data and moBRCA-net uses an attention-based neural network to integrate multiomics features.

**Figure 4.**
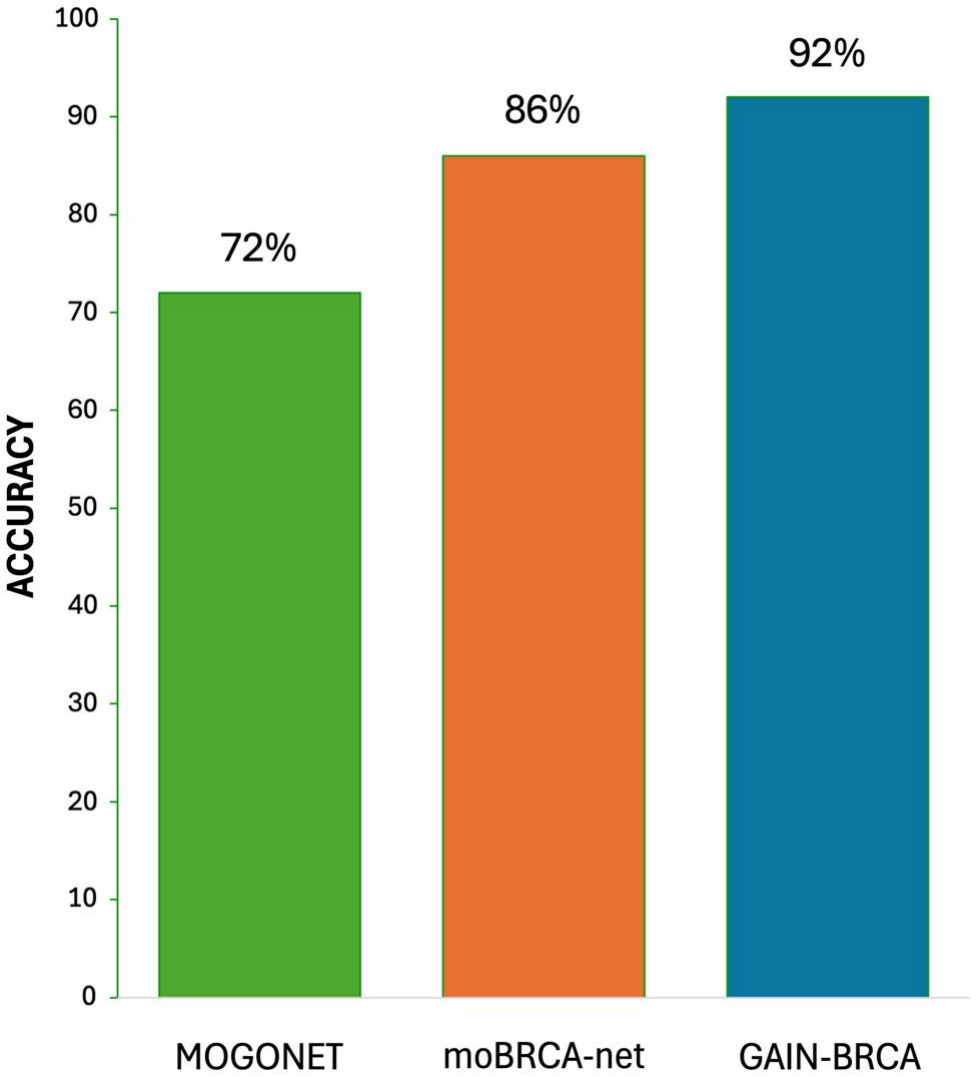
Comparison of graph-based multiomics integration models for breast cancer subtype classification. Bar plot comparing the accuracy of different graph-based multiomics integration approaches: MOGONET, moBRCA-net, and GAIN-BRCA.

Another method, HI-DFNForest uses a similar approach to our method, but we were unable to include this method in our comparison as its code is not publicly available. As seen in Fig. 4, GAIN-BRCA’s performance is exceedingly higher with an accuracy of 92% compared with those of moBRCA-net (86%) and MOGONET (72%). This comparison clearly demonstrates the advantage of our data integration and data augmentation approach to improve the prediction performance. Within the GAIN-BRCA framework, ANN performed the best over LR, RF, and KNN models in Luminal-A, Luminal-B, and HER2+ subtypes, achieving F1 scores of 0.95 for Luminal A, 0.8 for Luminal B and HER2+, and a perfect 1.0 for Basal with the highest overall AUROC of 0.98 and accuracy of 92%. Among the alternative models, RF also performed competitively in Basal subtype, reaching an F1 score of 0.95, indicating strong but slightly lower predictive power compared with ANN in that class. However, the performance of RF varied significantly across other subtypes, including an F1 score of just 0.37 for Luminal B and 0 for HER2+, highlighting the limitations in its generalizability. LR also showed notable improvement under GAIN-BRCA, achieving high precision and recall across Luminal A and Basal subtypes, while KNN showed moderate gains, although it remained less effective than the other models (Table 3). Despite these improvements, performance on the HER2+ subtype remained relatively low across all models, a trend likely driven by the underrepresentation of HER2+ samples (*n* = 38) compared with other subtypes such as Luminal A (*n* = 393), Luminal B (*n* = 124), and Basal (*n* = 114). Unbalanced datasets in machine learning result in improper data fitting of the models they generate. The data imbalance problem in data science is addressed by data augmentation, where the sample sizes of all classes are balanced by augmenting the sample size of smaller classes to match that of the largest class. In this study, we applied a data augmentation tool called ADASYN to balance the four breast cancer subtype datasets (Table 2).

### 3.8 GAIN-BRCA’s performance further improves with the augmented dataset

**Figure 5.**
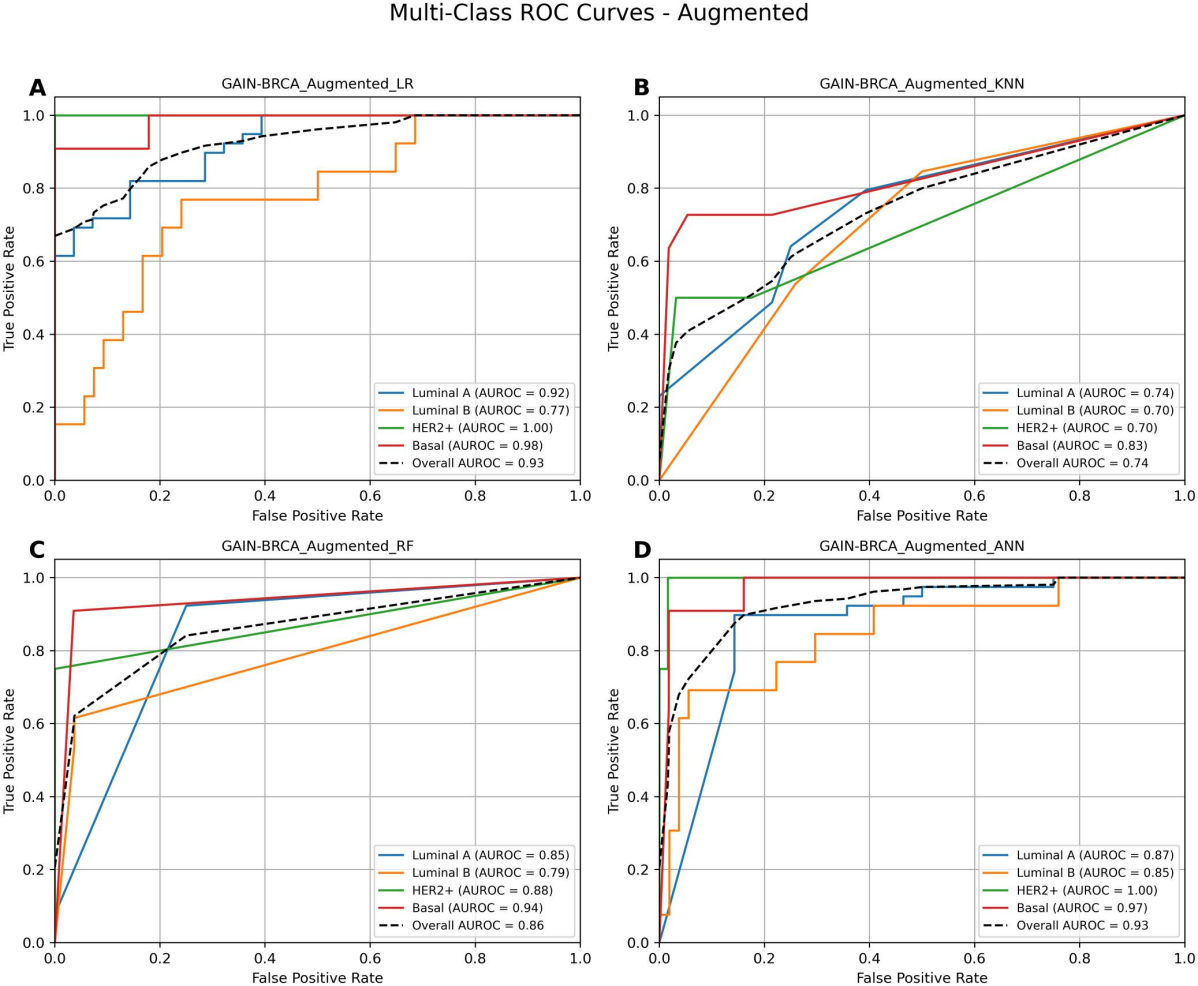
Performance of GAIN-BRCA models with data augmentation. This panel represents the ROC curves for the GAIN-BRCA augmented integration approach, using (A) logistic regression (LR), (B) K-nearest neighbor (KNN), (C) random forest (RF), and (D) artificial neural network (ANN). The augmentation shows strong performance across all subtypes and models. ANN and LR achieved the same AUROCs for HER2+ and almost the same for Basal (0.97 and 0.98, respectively). RF and KNN also show strong performance, especially for Basal, with KNN scoring slightly lower than other models but still showing a notable improvement compared to previous approaches. Overall AUC for the model as well as subtype-specific AUCs were presented.

Applying ANN on GAIN-BRCA to a class-balanced (augmented) dataset led to overall robustness and subtype level performance of several models, particularly those affected by class imbalance. ANN model on augmented dataset maintained a strong performance, achieving an AUROC of 0.93 and accuracy of 82%, with high F1 scores for HER2+ (0.8) and Basal (0.85) subtypes (Fig. 5D). LR and RF models achieved notable gains, reaching overall AUROCs of 0.93, and 0.86, and accuracy levels of 83.5% and 77.6%, respectively. KNN, which previously underperformed with imbalanced datasets, also improved to an AUROC of 0.74 and accuracy of 61.1%. These results underscore the effectiveness of data augmentation and class balancing to enhance both the robustness of the model and predictive accuracy. By addressing imbalances in the dataset and incorporating additional synthetic data, the models strengthen their generalization ability and establish a new performance benchmark in multi-omics classification.

### 3.9 Selection and functional characterization of important features (genes)

SHAP enables GAIN-BRCA to be an interpretable model by identifying gene features that contribute the most to prediction accuracy and enabling subtype-specific feature extraction based on importance scores. This model provides both general and class-specific insights, revealing distinct subtype associations that can guide downstream analysis. We have fit the GAIN-BRCA based transformed matrix into a random forest classifier and used the tool, *shap*. *Tree explainer*, retrieved 357 features, which were further analyzed to explore the unique biological roles of these subtype-specific genes. For each breast cancer subtype, the top SHAP-ranked genes may reveal potential biomarkers of tumor progression. Notably, the Luminal A and Luminal B subtypes show a common reliance on AURKA and CDC7, which are crucial for mitotic progression and linked to the maintenance of genomic stability. Overexpression of AURKA is associated with poor prognosis, particularly in Luminal A breast cancers (Ingebriktsen *et al.* 2024). The CDC7 gene is pivotal for DNA replication initiation and has been implicated in promoting cell proliferation in luminal subtypes, highlighting its potential as a therapeutic target (Rodriguez-Acebes *et al.* 2010). In contrast, PDSS1 and FOXM1 are exclusively linked to the Basal subtype, underscoring pathways associated with enhanced proliferation and aggressiveness characteristic of triple-negative breast cancers (TNBC) (Lu *et al.* 2018, Yu *et al.* 2021). Additionally, ESR1, prominently appearing in the HER2+ subtype, aligns with the hormonal nature of this group, suggesting its crucial role in mediating estrogen signaling. Variants in ESR1 have been associated with resistance to endocrine therapies, pointing to its significance as a therapeutic target in HER2+ positive breast cancer (Kuang *et al.* 2018). The presence of these genes underscores the complex interplay between hormonal and growth factor signaling in determining the biology of HER2+ tumors. Further examination reveals that DEPDC1B and MCM10 are identified across the Luminal A, Luminal B, and HER2+ subtypes, suggesting that cell cycle regulation and DNA replication processes are conserved mechanisms critical for the proliferation of these cancers. DEPDC1B has been implicated in promoting cell proliferation and tumor growth by regulating various oncogenic pathways, making it a potential therapeutic target in these subtypes (Wang *et al.* 2023). Similarly, MCM10, a component of the MCM complex involved in DNA replication, has been associated with tumorigenesis and poor prognosis in breast cancer, indicating its role in maintaining the replicative capacity of cancer cells (Murayama *et al.* 2021). In addition to identifying subtype-specific genes using SHAP, we carried out IPA to identify the upstream regulators (*n* = 1109) and downstream molecules (*n* = 3252) associated with these genes. This step is important to understand the functional significance of subtype-specific genes along with their upstream regulators, as well as downstream effectors. To understand the functional significance of genes in these three lists (SHAP features, upstream regulators, and downstream molecules), we used the SIFT and PolyPhen score filters to identify genes with observed deleterious or probably damaging mutations in the TCGA-BRCA cohort (Ng and Henikoff 2003, Flanagan *et al.* 2010). This filter narrowed the gene lists to 145 SHAP features, 257 upstream regulators, and 1565 downstream molecules and enabled us to obtain a more focused set of genes likely to influence breast cancer biology.

**Figure 6.**
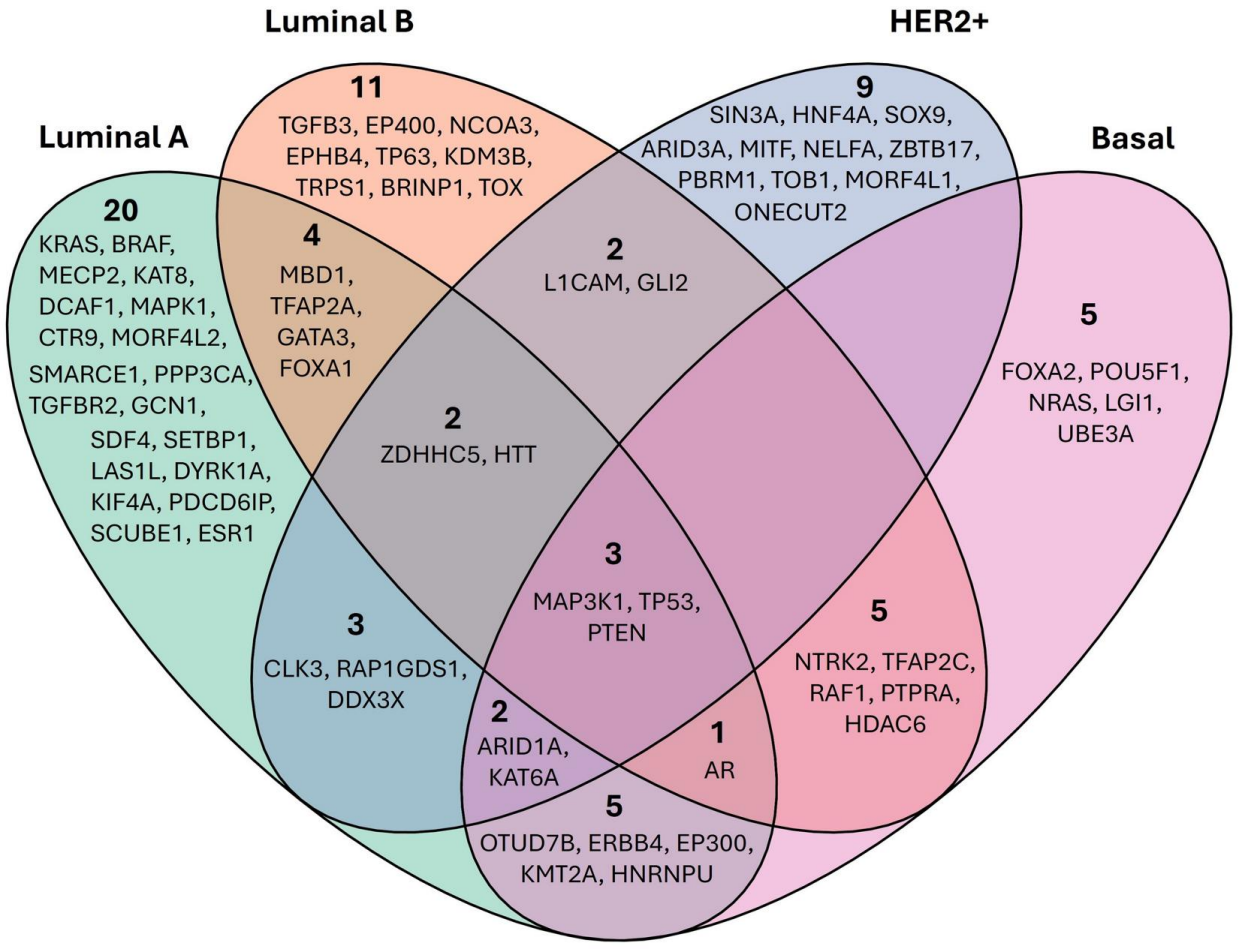
Venn diagram visualizing the distribution of genes across Luminal A, Luminal B, HER2+, and Basal breast cancer subtypes. The number of unique genes is mentioned with names for each subtype.

To further reduce the number of important features, we utilized the Database of Chromosomal Imbalance and Phenotype in Humans Using Ensembl Resources (DECIPHER) to extract haploinsufficiency scores (range from 0–1) for each gene, where 0 meaning there will be no effect on the phenotype and 1 meaning there will be an effect. We eliminated those with a score of 0 to ensure that observed mutations in these genes have a real effect on the phenotype (Bragin *et al.* 2014). This process yielded a combined list of 72 genes significantly associated with 176 patients, which were then categorized into the four breast cancer subtypes and visualized with a Venn diagram (Fig. 6). The Luminal A subtype included 48 genes, Luminal B contained 25 genes, while the HER2+ subtype had 23 genes, suggesting a more restricted set of potential biomarkers. The Basal subtype featured 21 genes, reflecting its distinct molecular profile. A Venn diagram was used to identify the unique gene list for each subtype (Fig. 6). We identified 20, 9, 11, and 5 unique genes in the Luminal A, Luminal B, HER2+, and Basal subtypes, respectively. PTEN, TP53, and MAP3K1 were shared across all subtypes. Notably, no genes were found to be common among the Luminal B, HER2+, and Basal subtypes.

### 3.10 Subtype-specific gene-survival associations

**Figure 7.**
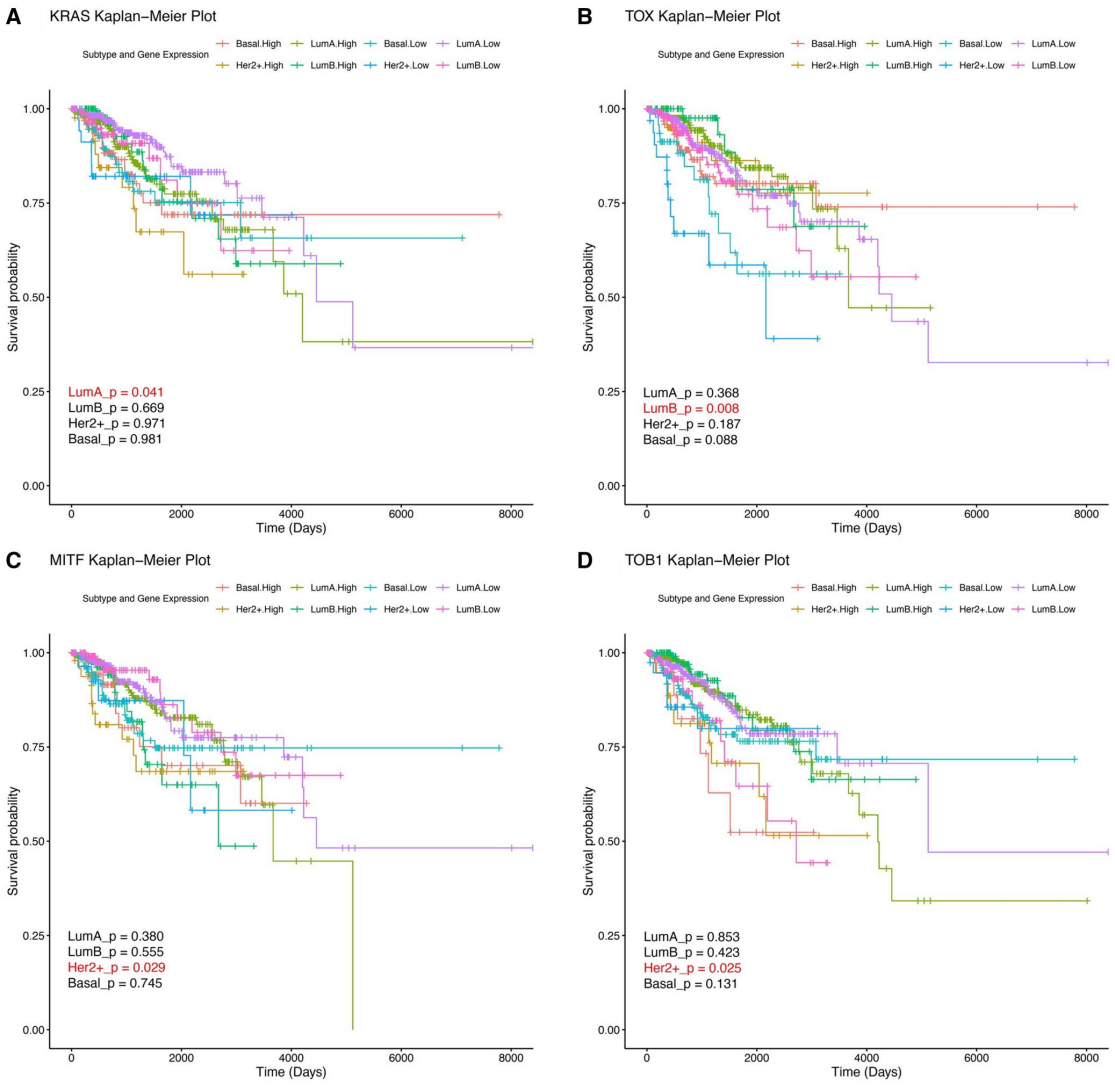
The subtype specific Kaplan-Meier survival plot demonstrating the impact of gene expression profile of (A) KRAS in Luminal A, (B) TOX in Luminal B, (C) MITF, and (D) TOB1 in HER2+ on overall survival of the patients across breast cancer subtypes.

Building upon the identification of unique gene sets for each breast cancer subtype (Fig. 6), we conducted a Kaplan-Meier (KM) survival analysis to evaluate the prognostic relevance of these subtype-specific genes in relation to overall patient survival in each subtype. Figure 7A illustrates a significant association between kirsten rat sarcoma **(**KRAS) expression and survival only in the Luminal A subtype (*P *= 0.041). Higher expression of KRAS is linked to worse survival outcomes in Luminal A, consistent with its known role in driving oncogenic pathways. This result aligns with previous studies suggesting KRAS as a prognostic marker in hormone receptor-positive breast cancers (Hwang *et al.* 2019). Figure 7B shows that thymocyte selection-associated HMG bOX **(**TOX) expression significantly impacts survival only in the Luminal B subtype (*P* value = 0.008) (Seksenyan *et al.* 2015). Luminal B tumors, typically more aggressive than Luminal A, may rely on pathways involving TOX, making it a potential therapeutic target for this subtype. Unique to the HER2+ subtype, two genes, microphthalmia-associated transcription factor **(**MITF) and transducer of ERBB2.1 **(**TOB1), were significantly associated with survival (*P* value = 0.029, and *P* value = 0.025), as shown in Fig. 7C and D, respectively. These results underscore the complexity of the HER2+ subtype, where multiple genes contribute to clinical outcomes. MITF, known for its role in melanocyte lineage and immune response, may offer new possibilities for immunotherapy in HER2+ patients (Ballotti *et al.* 2020). Similarly, TOB1’s correlation suggests its involvement in growth factor signaling pathways, pivotal for HER2+ tumor progression (Helms *et al.* 2009). However, in the Basal subtype, none of the five subtype-specific genes showed a significant correlation with survival. This finding reflects the intrinsic heterogeneity and triple-negative nature of Basal tumors, emphasizing the need for multigene or pathway-level approaches to uncover prognostic biomarkers in this aggressive subtype. These results highlight the importance of integrating multiomics data to unravel subtype-specific survival mechanisms. The strong subtype-specific correlations observed for KRAS, TOX, MITF, and TOB1 suggest their utility as both prognostic markers and therapeutic targets. Moreover, the lack of significant findings in the Basal subtype underscores the unmet need for novel biomarker discovery in triple-negative breast cancer. This analysis reinforces the potential for using molecular stratification to guide personalized treatment approaches, ultimately improving survival outcomes for breast cancer patients.

The GAIN-BRCA study is limited to only three omics: mRNA, miRNA, and DNA methylation, but it could be expanded to other omics data, such as DNA mutations and proteomics. However, with the addition of each omics modality, the computational cost will increase significantly due to the high dimensionality of features and correlations to be computed. This complexity, as highlighted in prior reviews (Zhang *et al.* 2022, Wekesa and Kimwele 2023), may limit scalability as the number of omics layers increases. Despite these challenges, advancements in next-generation software architectures (like CUDA) along with the hardware designed for multiplication of large matrices (like GPUs) hold promise for overcoming these barriers, enabling more efficient multiomics data integration.

## 4 Conclusions

The central idea of this study lies in the extraction and utilization of interdependencies among different omics features. While DNA methylation, miRNA, and mRNA represent different omics modalities in the cascade of gene regulation, mRNA levels (gene expression) are highly regulated with the first two. Due to these dependencies, each omics data cannot be treated independently. In this study, we developed GAIN-BRCA as a graph-based omics integration method that builds a weighted matrix, i.e. transformed feature representation for breast cancer subtype classification. These weights among different omics features (CpG-mRNA and miRNA-mRNA) have embedded biological correlations derived based on their relationships. GAIN-BRCA outperformed other neural network-based prediction models using concatenation and autoencoder approaches, as well as other popular methods, MOGONET and moBRCA for breast cancer subtype classification. SHAP prioritized the features for each subtype individually, which enabled us to limit our downstream analysis to the top-ranked features associated with breast cancer subtypes. KM survival analysis of genes uniquely identified in each subtype showed a significant correlation of certain genes with patient survival in all but the basal subtype. We believe that our novel graph-based multiomics data integration approach, coupled with explainable AI opens new avenues for cancer subtyping and the identification of subtype-specific therapeutic targets for precision medicine.

## Supplementary Material

vbaf116_Supplementary_Data

## Data Availability

The data underlying this article are available at https://zenodo.org/records/15175435.
