# Supplementary material for: GAIN-BRCA: a graph-based AI-net framework for breast cancer subtype classification using multiomics data
Source: Bioinform Adv. 2025 May 14;5(1):vbaf116. doi: 10.1093/bioadv/vbaf116 (PMC12151285; doi:10.1093/bioadv/vbaf116)
Supplement: vbaf116_Supplementary_Data [file vbaf116_supplementary_data.docx]

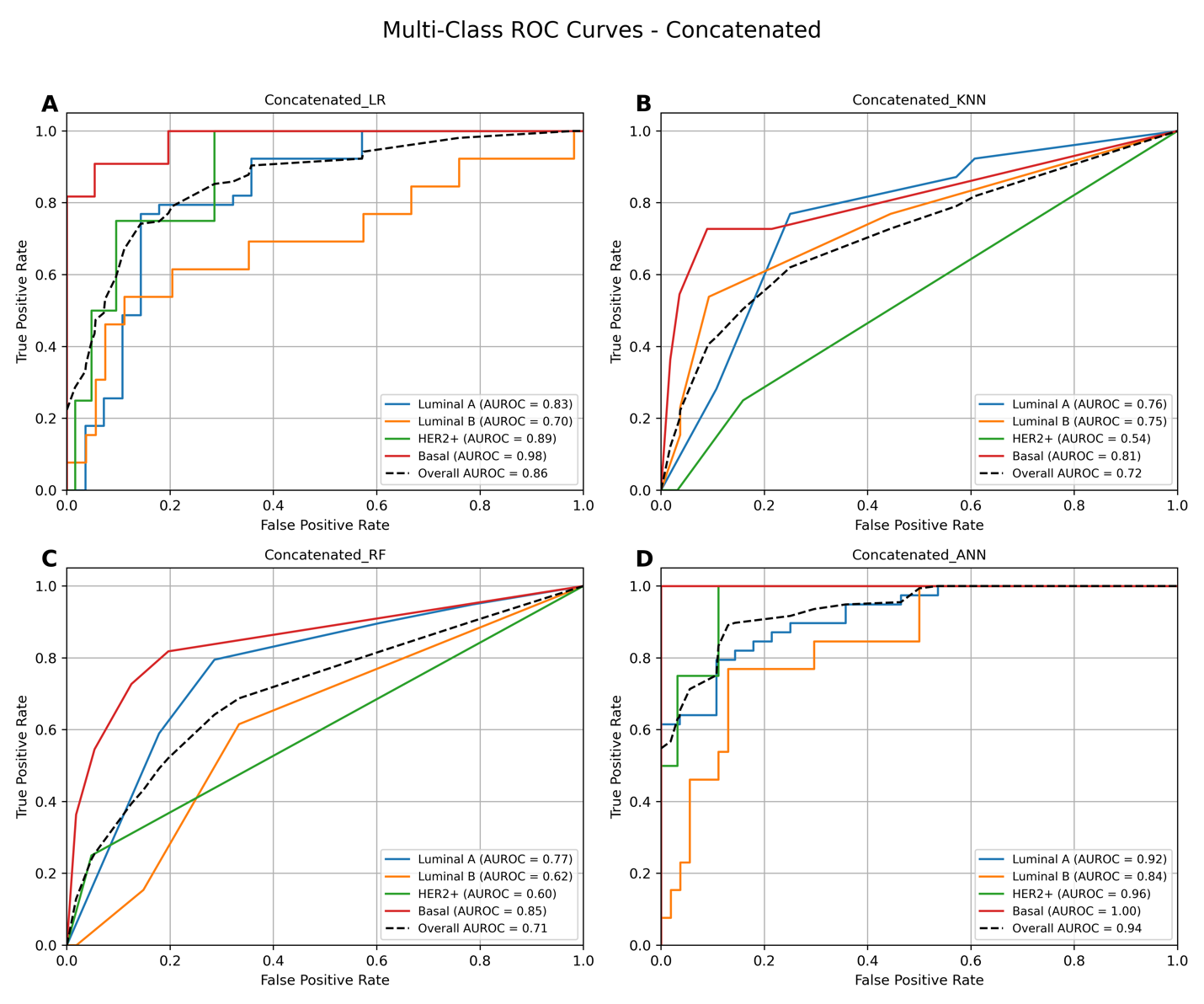


**Supplementary Figure 1:** Performance of concatenation based integration models. This panel illustrates the ROC curves for the Concatenation integration approach using (A) Logistic Regression (LR), (B) K-Nearest Neighbor (KNN), (C) Random Forest (RF), and (D) Artificial Neural Network (ANN). The overall AUROCs of LR and ANN are highest almost very close. Luminal A (blue) and Basal (red) show higher AUROC values across models, especially in ANN with AUROCs of 0.92 and 1.00, respectively. LR also performs well for the HER2+ subtype with an AUROC of 0.89. However, the models struggle with Luminal B, with lower AUROCs seen across all models, particularly in RF and KNN.


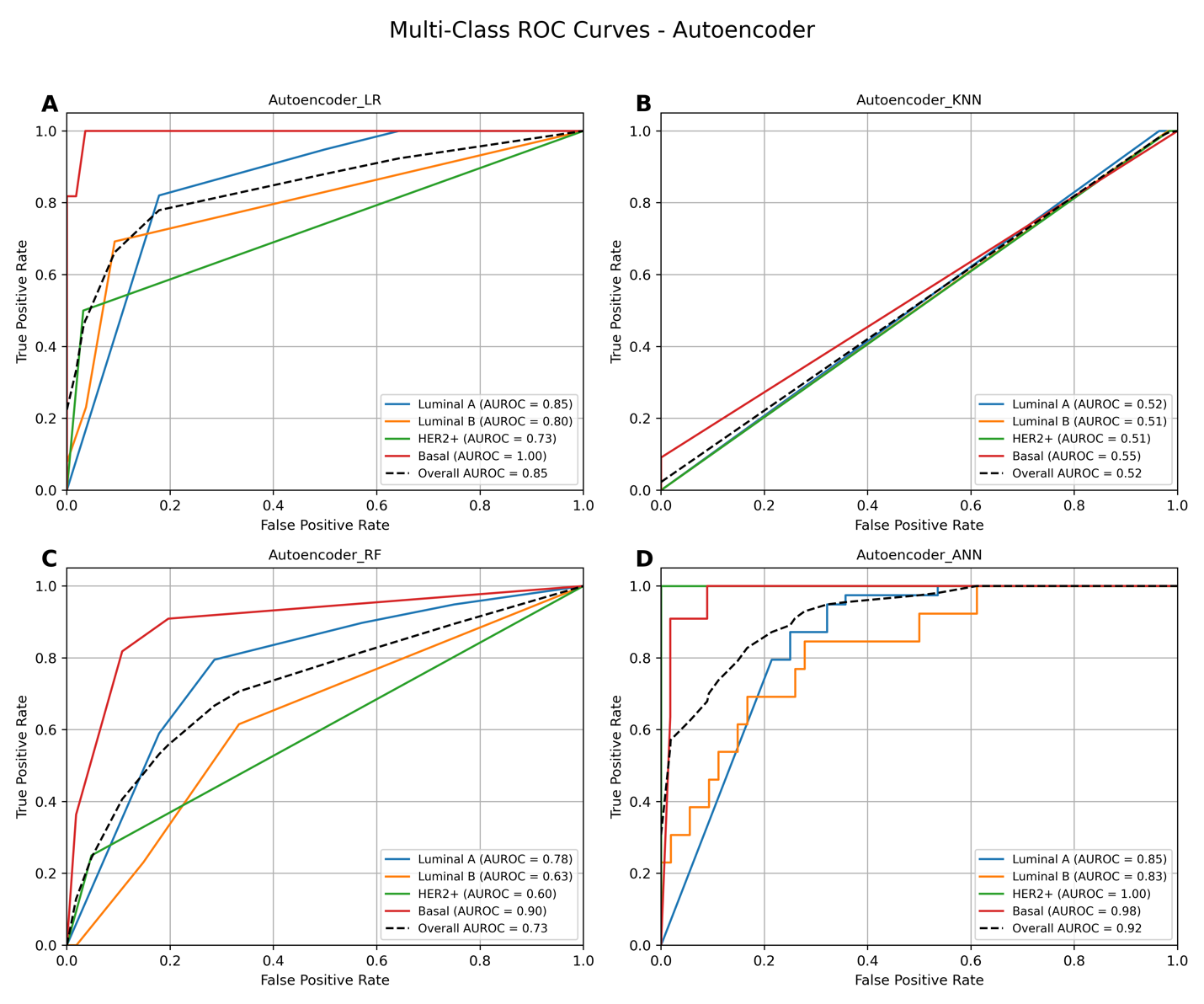


**Supplementary Figure 2:** Performance of Autoencoder based integration models. This panel shows the ROC curves for the Autoencoder integration approach using four machine learning models: (A) Logistic Regression (LR), (B) K-Nearest Neighbor (KNN), (C) Random Forest (RF), and (D) Artificial Neural Network (ANN). Each plot contains four ROC curves representing the following breast cancer subtypes: Luminal A (blue), Luminal B (orange), Her2+ (green), and Basal (red). The performance of LR and RF is notably higher for the Basal subtype, with an AUROC of 1.00 and 0.90, respectively, while other subtypes show lower AUROCs, especially for Luminal B and Her2+ across all models. Th overall AUROC of ANN is surpassed the all models followed by the LR.
